# Editorial: Mechanisms and interventions for enhancing cognitive reserve in aging populations

**DOI:** 10.3389/fnins.2026.1830725

**Published:** 2026-04-28

**Authors:** Juan Silva-Pereyra, Thalía Fernández, Valia Rodríguez-Rodríguez

**Affiliations:** 1 Facultad de Estudios Superiores Iztacala, Universidad Nacional Autónoma de México, Tlalnepantla, Estado de México, Mexico; 2 Departamento de Neurobiología Conductual y Cognitiva, Instituto de Neurobiología, Universidad Nacional Autónoma de México, Juriquilla, Querétaro, México; 3 College of Health and Clinical Sciences, Aston University, Birmingham, United Kingdom; 4 Faculty of Science and Technology, Middlesex University, London, United Kingdom

**Keywords:** brain electrical activity, brain imaging, cognitive processes, cognitive reserve, healthy aging, internet use, leisure activity, physical activity

Population aging, driven by increased life expectancy, has transformed global health needs. Preserving cognitive function in middle and older age is today a critical health priority, as advancing age is the principal non-modifiable risk factor for neurocognitive disorders (NCDs) (Colita et al., 2024; Whalley et al., 2004). Aging implies biological, psychological, and social changes, and entails a physiological cognitive decline that, in some cases, can be exacerbated by pathological processes leading to NCDs. In this context, cognitive reserve (CR)—a multidimensional construct that reflects cognitive stimulation throughout life—acts as a protective resource that helps older adults remain resilient to cognitive decline (Stern, 2002, 2009).

The concept of CR refers to the brain's capacity to maintain adequate cognitive function despite aging or neuropathological damage. This capacity would explain why some people show fewer clinical signs of cognitive impairment or dementia than others, even when they have comparable levels of brain pathology. Traditionally, CR has been estimated using proxies such as educational attainment, cognitive engagement, premorbid intellectual ability, occupational complexity, leisure pursuits, and physical activity, modulated by genetic or neurophysiological factors (D'Aurizio et al., 2023; Scarmeas and Stern, 2003; Wilson et al., 2019). The collection of studies that we present in this number aims to examine, from cross-sectional and longitudinal perspectives, how these CR variables influence cognitive performance and the trajectories of brain aging.

For instance, Ottaviani et al. analyzed the association between CR subdomains and cognitive status, while controlling for sex and age, in a sample of 317 older adults with subjective cognitive impairment, mild cognitive impairment (MCI), and Alzheimer's or mixed dementias. Using the Cognitive Reserve Index (CRIq) and stratifying participants by sex and dementia stage, the authors found that leisure activities were the main determinant of cognitive status. Unlike education and occupation, engagement in leisure activities is a dynamic and potentially modifiable factor across the lifespan, underscoring its relevance as a target for preventing neurocognitive disorders. It also raises the question of whether current methods for assessing cognitive function need to be refined. However, the authors note that the monocentric design of their study may limit the generalizability of the findings.

Related to this dynamic domain, Zhou et al. examine social participation in a large sample of 17,962 adults aged 45 and older and report that higher levels of social participation were associated with better cognitive functioning, a relationship that seemed to be indirectly enhanced by grandchildren caregiving. However, the mediator effect of grandchildren caring was modest, and several unexamined factors limit the interpretation of this result. For instance, the caregiving role may function as a custodial burden rather than a voluntary, enriching activity, but it was not explored. Additionally, potential sex differences were not considered: grandfathers may experience childcare as discretionary engagement, whereas grandmothers often face more intensive custodial expectations that can lead to stress and fatigue. These overlooked elements could have influenced the observed relationship between caregiving and cognition. The study also found that the positive relationship between social participation and cognition was attenuated by the presence of depression. Accordingly, the authors emphasize that public policies should prioritize depression-reduction strategies, including forms of social engaging tailored to age, income and residential settings, as a means of supporting cognitive health in aging populations.

Another central domain of CR is physical activity, addressed from multiple perspectives in the compiled articles. Shi et al. examined physical activity as a proxy for cognitive impairment, analyzing the combined impact of exercise in adolescence and old age on mild cognitive impairment (MCI), also known as mild NCD. In a sample of 1,615 older adults, they observed that those who exercised in both life stages had a lower risk of minor neurocognitive impairment compared to those who did not exercise in either stage. However, no significant differences were found in regional brain volumes or in biochemical markers associated with cognition, such as BDNF, IGF-1, or homocysteine. These results suggest that physical activity throughout life promotes healthy cognitive aging, although the underlying neurobiological mechanisms remain unclear. It is important to note that Shi et al. used a cross-sectional design and self-reported data, which may introduce recall bias regarding adolescent habits. In addition, their dichotomous assessment of exercise prevented examination of possible dose-response effects. The study was also conducted within a single Japanese region, which limits the generalizability of the findings, and the definition of “old age” as ≥65 years may overlook earlier stages of cognitive decline.

From a more everyday perspective, Sanchez-Lopez et al. focused on how incidental physical activity (IPA) related to older adults' ability to inhibit automatic responses. IPA refers to the routine, unplanned movements that occur in daily life such as walking or performing household chores. Behavioral results confirmed the presence of Stroop effect irrespective of IPA level, but the effect of the difference between conditions (congruent and incongruent) did not differ between groups. However, event-related brain potentials recorded during the task execution showed that individuals with higher IPA exhibited greater late negativity, suggesting more efficient inhibitory processing at later task stages. This suggests that IPA may enhance executive functioning at the neural level, even when behavioral effects are not evident, and that its accessibility makes it a valuable strategy for supporting cognitive function.

Complementarily, Wang et al. analyzed the relationship between the diversity in the devices used to access the internet—referred to as “internet use”—physical activity, and cognitive performance in 2,383 older adults. They found that both the diversity and physical activity were positively associated with cognition, and that physical activity mediated the relationship between internet use diversity and cognitive performance. This finding highlights the complexity of lifestyle factors and their interaction in maintaining cognitive function. It is important to note that, in examining internet use, the authors did not control for income level; instead, they dichotomized the variable into “income” vs. “no income.” This omission is important because ownership of multiple devices is more indicative of financial capacity than of cognitive ability or executive functioning. Given that wealth strongly influences health, education, occupation, and thus cognitive status, failing to control for it limits the interpretability of their findings.

From a broader perspective, Sun et al. conducted a meta-analysis of 55 prior meta-analyses to identify which types of physical activity are most beneficial for different pathologies. However, they point out that this umbrella review was constrained by heterogeneity across primary meta-analyses, publication bias, overlapping studies, inconsistent diagnostic criteria, and exclusion of non-English literature. The study concluded that there is moderate-to-high-quality evidence supporting Exergaming, Tai Chi, and traditional Chinese exercises for improving cognition in MCI; resistance training and Tai Chi for dementia; aerobic exercise for Alzheimer's disease; mind-body exercises for Parkinson's disease; and multicomponent exercises for post-stroke cognitive decline.

Beyond behavioral factors, neurobiological mechanisms of CR have been proposed. Schwarz et al. explored redundancy in brain network functional connectivity (BFR) as a potential CR metric. In a sample of 301 healthy participants aged 18–89, they observed that BFR specifically modulates the relationship between cortical thickness and episodic memory, and that higher BFR is, counterintuitively, linked to poorer episodic performance. These results suggest that BFR may reflect compensatory mechanisms specific to certain cognitive domains, which limit its utility as a broad metric of CR. The study does not clearly determine whether high BFR represents a beneficial reserve capacity or a compensatory response to underlying pathology.

Similarly, several studies have examined resting-state electrical brain activity as an indicator of compensatory mechanism or as a predictor of response to interventions. Perez et al. compared older adults with high and low CR and found no neuropsychological differences; however, individuals with low CR showed greater delta and theta power in the resting EEG, which was interpreted as a possible compensatory mechanism to sustain cognitive function. However, most study participants had higher-than-average cognitive levels, which may have reduced the variability in CR and masked potential neuropsychological differences. On the other hand, Juras et al. demonstrated that specific patterns of parietal alpha and resting-state theta activity can predict the magnitude of cognitive improvement after training, suggesting that EEG may help personalize cognitive interventions. Yet the study was limited by a modest sample size focused solely on middle-aged adults, the absence of a control group to determine whether EEG changes were training-specific, and a short follow-up without real-world outcome measures.

Finally, the need for longitudinal clinical trials that integrate multiple domains of CR is increasingly emphasized. In this direction, Bherer et al. proposed a 12-month intervention combining physical and cognitive training in older adults with cardiovascular risk factors. This design will enable the evaluation of cognitive effects alongside cerebrovascular mechanisms and potentially mediating biomarkers, contributing to a more comprehensive understanding of how lifestyle factors shape CR and healthy aging.

Two limitations stand out in this topic research. First, most of the included studies use a cross-sectional design. This design could reduce the ability to establish causal relationships between different indicators of cognitive reserve and cognitive performance. Second, there is potential bias in self-reported measures (Sanchez-Lopez et al.; Shi et al.). Many studies draw samples from specific regions of the world, which may compromise generalizability. Some authors, such as Sanchez-Lopez et al., imposed very strict inclusion criteria. They aimed to avoid confusion about the relationship between cognitive reserve indicators and brain function, thereby enabling internally valid results. Although this strategy initially limits generalizability, it may lay the groundwork for future research with broader and more diverse samples.
